# CXCL17: The Black Sheep in the Chemokine Flock

**DOI:** 10.3389/fimmu.2021.712897

**Published:** 2021-07-15

**Authors:** Stepan S. Denisov

**Affiliations:** Department of Biochemistry, Cardiovascular Research Institute Maastricht (CARIM), University of Maastricht, Maastricht, Netherlands

**Keywords:** chemokines, CXCL17, DMC, VCC-1, CXCL8

## Introduction

Chemokines (*chemo*tactic cyto*kines*) are a class of small secreted proteins that regulate chemotaxis and control cell trafficking. These proteins are structurally conserved and adopt a so-called chemokine structural fold with a flexible N-terminus and N-loop followed by three-stranded antiparallel β-sheet and a C-terminal α-helix (1). This three-dimensional fold is usually stabilized with four cysteines that form two disulfide bonds. All chemokines fall into one of the two major (CC and CXC) or the two minor (XC and CX3C) groups according to the position of the first two cysteine residues in the amino acid sequence. Up to date, 43 chemokines, not counting isoforms, are known in human (2). The latest addition to the chemokine family is mucosal CXC-type chemokine CXCL17 (3). Although the body of literature about CXCL17 is growing and now includes several dozens of published articles focusing on or referring to it, belonging of this protein to the chemokine family remains debatable.

## CXCL17 Discovery

The original sequence of the protein in question has been identified as a part of the large-scale Secreted Protein Discovery Initiative (SPDI) and submitted to GenBank with the accession code AY358433 under the name DMC (4). DMC contains 119 amino acid residues, including six cysteines, and its function could not be attributed through homology-based methods by then. Subsequently, the activity of DMC as a chemoattractant of monocytes and dendritic cells was demonstrated (5). Moreover, the fold recognition algorithm ProHit has been used to elucidate the DMC structure. Similarity with the chemokine fold of CXCL8 (IL-8) has been found and proved by circular dichroism (CD) and secondary structure prediction tools. Independent of the studies described above, the same protein and its murine analog have been identified using tumor transcriptional microarray analysis and has been named VCC-1 (6). VCC-1 has been attributed to CXC-type chemokines based on sequence homology with CCL16 (SCYA16) and CCL17 (SCYA17). The next piece of the puzzle is provided in (7), where posttranslational cleavage of the 6-Cys to a mature 4-Cys protein has been shown. After that, the term CXCL17 has been consolidated and further used in numerous follow-up studies.

## Old Claims Revisited

CXCL17 has been classified as a CXC-type chemokine in two independent research articles (5, 6). In the former (6) the claim is based on the observation that “the human [CXCL17] sequence … contains six cysteine residues, with four of them occurring in two CXC motifs”. However, presence of the CXC motif is necessary, but not sufficient for a protein to be attributed to CXC-type chemokines. After assigning VCC-1 to the CXC-type chemokines, the authors supported this notion by analysis using a linear hidden Markov model (HMM) to identify closely related proteins. Interestingly, only two CC-type chemokines were found as homologs whereas one would expect CXC-type chemokines in this case. This approach and the results obtained do not provide sufficient evidence to classify VCC-1 as CXC-type chemokine.

**Figure 1:**
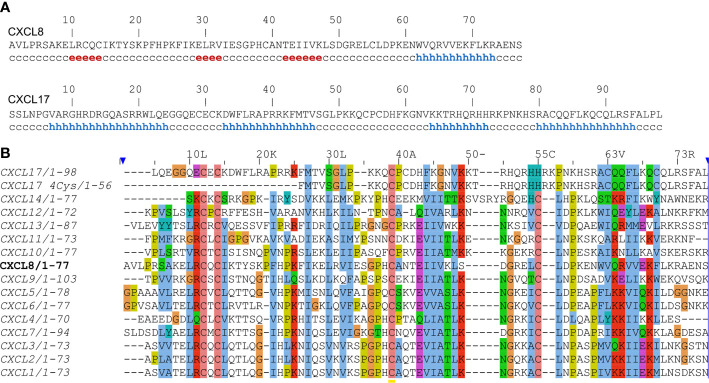
**(A)** Secondary structure prediction of human CXCL8 and CXCL17 using DSC webserver. Residues involved in β-strands and α-helix are shown by ‘e’ and ‘h’, respectively. **(B)** Sequence alignment of human CXC-type chemokines using the Clustal Omega webserver. The sequence of CXCL8 is set as a reference.

The second paper (5) elaborated on the structural analysis of CXCL17 and attributed CXCL17 to the CXCL8-like structural motif using ProHit algorithm. The authors supported this notion by “secondary structure prediction performed for DMC sequence (PHD, DSC; data not shown) [which] are in agreement with an IL-8-like fold”. Remarkably, when DSC server (8) was used during preparation of this opinion, only α-helical regions are predicted for the CXCL17 sequence, in contrast to the CXCL8 sequence, analysis of which yielded the canonical chemokine secondary structure with three β-strands and one α-helix (**Figure 1A**). Further authors of the study in question have used circular dichroism to compare DMC and CCL5. The spectra for both proteins have single minimum at ~200 nm. Despite the visible hypsochromic shift of the DMC spectrum compared to the CCL5 one, the authors concluded that DMC is structurally similar to CCL5. CCL5 is known to form high order oligomers and even fibrils (9) which could affect the CD signal. Therefore, comparison with CCL5 could be incorrect and in that case the fairer reference would be CXCL8 itself as authors claimed “an IL-8-like fold” of DMC. However, published CD spectra of CXCL8 have two minima at 222 and 208 nm and a maximum at 190 nm (10), which is unrelated to the published DMC spectrum.

Moreover, there is a discrepancy in the disulfide bond connectivity of the six cysteine residues of CXCL17. In one study (5) [C1-C3; C2-C4] linkage is derived from DMC sequence alignment with CXCL8_E38C/C50A_. However, this contradicts later work (7), where the mature CXCL17 does not contain the first couple of cysteines. From this observation, [C3-C5; C4-C6] linkage is hypothesized based on the structure of monomeric CXCL8. Sequence alignment (**Figure 1B**) demonstrated that 4-Cys CXCL17 does not follow the typical CXC-type cysteine pattern. In addition, it is simply too short to adopt a chemokine fold.

## Discussion

Chemokines, regulate chemotaxis and play a crucial role in numerous (patho)physiological processes. Despite this, chemokines are primarily assigned by their structure and not by function. While CXCL17 undoubtedly has a chemotactic activity, arguments for its belonging to the chemokine family do not hold up from a structural point of view, as I have attempted to demonstrate above. What could be considered as a technical nuance of protein nomenclature, is actually having a significant impact on research and could further mislead researchers in an already complex field of chemokine signaling. For example, CXCL17 has been used in chemokine heterodimerization study (11). Ongoing debates about CXCL17 receptor(s) is influenced as well and a putative candidate GPR35 is already named CXCR8 in several studies and reviews (3). Surprising research inertia could be observed in the recently published paper (12) in which the authors modeled CXCL17. After obtaining the structure with four α-helixes, which has nothing in common with chemokines, results have been discussed in terms of chemokines anyway.

The opinion presented by no means undermines or challenges biological results obtained over more than a decade of CXCL17 research. It simply implies that nomenclature must be revisited. *Ceterum autem censeo*, CXCL17 should be renamed.

## Author Contributions

SD has written this opinion. The author contributed to the article and approved the submitted version.

## Funding

This work was supported by the Maastricht Universitair Medisch Centrum.

## Conflict of Interest

The author declares that the research was conducted in the absence of any commercial or financial relationships that could be construed as a potential conflict of interest.
